# State-resolved studies of CO_2_ sticking to CO_2_ ice

**DOI:** 10.3389/fchem.2023.1250711

**Published:** 2023-08-24

**Authors:** Charlotte Jansen, Ludo B. F. Juurlink

**Affiliations:** Leiden Institute of Chemistry, Leiden University, Leiden, Netherlands

**Keywords:** CO_2_, nu3, state resolved, molecular beam, condensation

## Abstract

Internal vibrations may affect the adsorption, scattering, and reactions of molecules impinging onto a surface. The energy of the *ν*_3_ antisymmetric stretch vibration of CO_2_ slightly exceeds the desorption energy of CO_2_ bound to CO_2_ ice. We use supersonic molecular beam techniques and rovibrationally state-resolved excitation to determine whether this vibration affects condensation of gas phase CO_2_ to its ice. We detect sticking and CO_2_ ice formation using RAIRS and quantify the sticking probability using the King and Wells method with modulation of the vibrational excitation and Fourier transform based detection. We find that the influence of this vibration on the structure of the formed ice and on the sticking probability is negligible under our conditions. Based on our detection limit, we quantify the weighted average sticking probability at approximately 0.9 and the difference between the state-resolved and weighted average sticking probability as below 0.5%.

## 1 Introduction

Collisions of CO_2_ with surfaces are of interest to a broad range of scientific fields. In astronomy and astrochemistry, CO_2_ occurs, a. o., in the interstellar medium (ISM) (d’Hendecourt and Jourdain de Muizon, 1989), both in gaseous and ice phases (Van Dishoeck, 2004), and in comets (Despois et al., 2005). In the chemical industry, CO_2_ is a major reactant in the Cu/ZnO-based catalytic production of methanol (Rostrup-Nielsen, 1984). Collisions of CO_2_ with surfaces are generally not reactive in a direct sense (Burghaus, 2014). Molecular sticking to a surface likely precedes subsequent chemical processes, especially at low-temperature conditions, such as those prevalent in interstellar space.

Molecular vibrations may affect the adsorption, desorption, and reactions of molecules to surfaces. Sibener and Lee (Sibener and Lee, 1994) found that internal vibrations of SF_6_ and CCl_4_ had a significant impact on the adsorption and reflection of these gas-phase molecules onto their bulk solids at low collision energies. In precursor-mediated reaction of CH_4_ on Ir (111) (Dombrowski et al., 2015) and SiH_4_ on Si(100) (Bisson et al., 2008), internal vibrations were found to enhance dissociative adsorption. A study of NO’s trapping probability on Au (111) found, on the contrary, no effect of vibrational excitation (Wodtke et al., 2005).

For CO_2_, a study by Weida et al. (Weida et al., 1996) investigated CO_2_ desorption from crystalline CO_2_ ice at varying temperatures, hence varying desorption rates, using state-selected detection. They found no evidence of an effect on the desorption by the molecules’ vibrations, rotations and even orientations. From detailed balance, they argued that if there is no preference for a certain quantum state for desorption, neither will there be for the process of adsorption. However, due to the limitations of their methods, only the *ν*_2_ bend vibration in CO_2_ was studied. Its vibrational energy (667 cm^−1^ or 8.0 kJ/mol) is well below the desorption barrier, the sublimation energy being approximately 26 kJ/mol. The *ν*_3_ fundamental vibrational mode (2,349 cm^−1^ or 28.1 kJ/mol) slightly exceeds the latter. A more recent study by Ioppolo et al. (Ioppolo et al., 2022) found restructuring and minor desorption of amorphous CO_2_ ice after irradiation with resonant IR light (*ν*_2_ and *ν*_3_). For crystalline ice, they found no restructuring, but possible desorption. Their results were inconclusive on vibrational effects to desorption.

Here, we study the effect of vibrational excitation (*ν*_3_) on the adsorption of CO_2_ onto CO_2_ ice grown on a Cu(111) surface at 80 K. We excite the *ν*_3_ asymmetric stretch of CO_2_ molecules in a molecular beam with light from a tunable continuous wave (cw)-infrared (IR) laser. We look for any changes in the sticking probability of the CO_2_ by modulating the excitation and using a Fourier transform based detection scheme. An effect of vibrational excitation on either the adsorption or the desorption rate will change the effective sticking probability. We also monitor the surface with Reflection Absorption IR Spectroscopy (RAIRS) and look for any structural differences in the spectra of the resulting CO_2_ ice layer with and without the additional vibrational excitation. At 80 K, the ice will be crystalline. Ioppolo et al. found no restructuring after IR irradiation of crystalline CO_2_ ice. However, our situation may be different, as they irradiated the CO_2_ ice after deposition, while we resonantly excite vibrations in the gaseous CO_2_ molecules prior to adsorption.

## 2 Experimental

**FIGURE 1:**
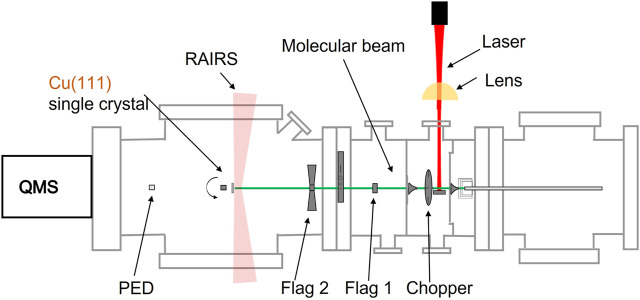
Schematic representation of the apparatus, consisting of 4 connected and differentially pumped vacuum chambers through which a molecular beam travel from right to left, the light from an excitation laser (red) and lens (yellow), a fast chopper wheel, a Cu(111) single crystal held on an x, y, z, *θ* manipulator in the UHV chamber of the system, a room temperature bolometer (PED), two quadrupole mass spectrometers (QMS), and RAIRS capability.

Our experiments are performed using a home-built ultra-high vacuum (UHV) system, that has been used for non-state-resolved reaction dynamics measurements before (Cao et al., 2018; van Lent et al., 2019; Jansen and Juurlink, 2021). It consists of an analysis chamber with a base pressure of 2 × 10^−10^ mbar and a series of differentially pumped chambers for creation of a molecular beam. Figure 1 schematically illustrates the apparatus. The molecular beam is shaped by various skimmers that separate differential pumping stages and doses CO_2_ onto a Cu (111) single crystal in the UHV chamber.

The molecular beam is created by expanding high-purity CO_2_ (Linde Gas, purity 5.3) at 300 K. A flow controller feeds the gas at 4 bar into a vacuum chamber ( $<1\times 10^{-3}$ mbar) through a 28 *μ*m circular orifice in a tungsten nozzle. Two skimmers and a third orifice select only the center part of the expansion, resulting in a molecular beam with an angular spread of less than 1°. During the expansion, kinetic and rotational energies are converted to translational energy along the beam axis, resulting in an average beam velocity of approximately 587 m/s as determined by time-of-flight (TOF) spectrometry (see Supplementary Material S1). The rotational temperature within the beam is cooled to approximately 26 K [see ref (Jansen et al., 2023)].

The molecular beam travels from the source chamber through two differentially pumped vacuum chambers and finally enters the UHV analysis chamber. In the first of these two chambers, a high-speed chopper wheel allows us to create short gas pulses from the continuous gas expansion for TOF spectrometry. The wheel can be stopped in the ‘open’ position. In the second of these chambers, a beam stopper can block the beam from hitting a sliding beam valve that separates this chamber from the UHV analysis chamber. The sliding beam valve contains various openings and controls the size and shape of the beam impinging onto the sample, a room temperature bolometer (pyro-electric detector, PED)) and the ionizer of the QMS used for TOF measurements.

On the beam axis in the analysis chamber, the edge of a disc can be rotated by an UHV-compatible stepper motor. The disc’s edge is manufactured to have two identical ‘open’ and two ‘closed’ sections, each of 90°. The closed sections have a slanted edge as not to reflect the molecular beam back into the last of the differentially pumped chambers. Combined with the beam stopper in the differential stage, this disc allows us to accurately perform King and Wells-type sticking probability measurements (King and Wells, 1972), but using a modulation technique as described below. We will refer to the beam blocker and the disc as the two beam flags from here onward.

The Cu single crystal is suspended from a copper block at the end of a vertical ‘cold finger’, i.e., a hollow tube protruding through an x, y, z, *θ* manipulator. The crystal is cooled by pouring liquid nitrogen (LN2) into the tube. It is kept at a constant temperature of 80 ± 1 K during experiments, as measured by a K-type thermocouple laser welded to the crystal’s edge. Adsorption of CO_2_ onto the sample is monitored using an FTIR (Bruker Vertex 70) with an external LN2-cooled MCT detector to perform RAIRS during the experiments. The IR beam paths outside of the UHV chamber, including internal parts of the FTIR, are purged continuously with gaseous dry N_2_ from an in-house system fueled by the exhaust from the local NMR facility.

The CO_2_ *ν*_3_ antisymmetric stretch vibration is excited in the molecular beam with laser light from an OPO (Argos model 2,400, module D), which we will from now on simply refer to as the laser. Its output can be tuned and is stabilized on a CO_2_ absorption frequency using the lamb dip measured in a reference cell. The laser light crosses the molecular beam a few cm after the initial expansion in the apparatus and 37 cm before molecules hit the Cu crystal. For this experiment, we tune the laser to the *R* (4) transition at 4,249.99 nm, where CO_2_ molecules are excited from the *v =* 0, *J* = 4 state to the *v* = 1, *J* = 5 state. This transition was chosen because *J* = 4 has the highest population at the rotational temperature of the molecular beam [see (Jansen et al., 2023)]. A cylindrical lens in the path of the laser is used to achieve rapid adiabatic passage (Jansen et al., 2023; Chadwick et al., 2014), resulting in nearly full (80%) population inversion. Considering the fractional population of the ground rotational state and the level of saturation, we excite approximately 15% of the CO_2_ molecules in the molecular beam. Herein, we neglect the fraction of vibrationally excited states nascent to the molecular beam. A moveable room-temperature bolometer (pyro-electric detector, Eltec model 406) in the UHV chamber is used to quantify IR power absorption by CO_2_ molecules on the molecular beam axis. All laser beam paths outside of the UHV chamber, including the entire OPO and optical components required for frequency detection and stabilization, are enclosed and purged continuously by dry gaseous N_2_. The > 400 ppm concentration of CO_2_ in air otherwise destabilizes the OPO and results in large power losses through absorption in the 1–2 m beam path between the laser and the UHV chamber.

At the start of an experiment, the Cu crystal is flashed to 300 K to remove any buildup of CO_2_, CO and H_2_O. As we do not regularly perform extensive sputtering and annealing cycles for these experiments, the surface should not be considered an atomically clean Cu(111) surface. It is most likely partially oxidized, as studied previously (Zhang et al., 2023). When the crystal has cooled to 80 K, a RAIRS background spectrum is taken. Subsequently, the surface is continuously monitored with RAIRS while CO_2_ is dosed from the molecular beam. The CO_2_ flux is estimated to be 0.15 ML/s as referenced to the Cu atom surface density for Cu(111). The experiment is repeated multiple times with the laser tuned to the CO_2_ excitation frequency and several times tuned to a non-resonant frequency as a control experiment. Between each experiment, the crystal is flashed to 300 K.

As described below, attempts to obtain information on the effective state-resolved sticking probability of CO_2_ by analyzing IR peak integrals was unsuccessful. The integrals increase with the CO_2_ dose time but did not allow us to detect variations in ‘laser on’ and’ laser off’ experiments. Hence, we measure the sticking probability of CO_2_ onto CO_2_ ice at 80 K with a modulated version of the King and Wells method (King and Wells, 1972) and a quadrupole mass spectrometer (QMS, Pfeiffer QMA 200) tuned to m/z 44.

A sticking probability experiment starts with a CO_2_ ice being prepared by continuously dosing CO_2_ onto the Cu crystal while being monitored with RAIRS. When the RAIRS spectrum shows sufficient intensity of the CO_2_ absorption line, the King & Wells experiment is started by closing both molecular beam flags. The first flag is reopened and the beam enters the UHV chamber, but is blocked by the second flag. This causes a rise in the CO_2_ partial pressure in the vacuum chamber, which is continuously monitored with the QMS. When the second flag is reopened, the beam impinges again onto the CO_2_ ice. The fraction of CO_2_ molecules that is adsorbed on the surface is removed from the background pressure in the chamber, resulting in a drop in the total pressure. The effective sticking probability is the ratio of the pressure drop after the beam impinges onto the ice and the pressure rise when the beam enters the chamber.

Note that the rate of desorption of CO_2_ at 80 K from the CO_2_ ice film is not affected by the molecular beam impinging onto the sample as the ice is many layers thick. The contribution of CO_2_ evaporation from the film to the background pressure is unaltered when the beam hits the sample. Hence, our measurement solely detects if the effective sticking coefficient, i.e., the net condensation of CO_2_ molecules onto the ice from the impinging beam, depends on the vibrational state population of that beam. It is only the latter that we alter by turning the laser ‘on’ and ‘off’. In the laser ‘on’ case, the heavily populated vibrationally excited state may adsorb or scatter more effectively in a direct sense than the vibrational ground state does. In case a vibrationally excited molecule adsorbs, it may also desorb again on a time scale much shorter than the experiment by V → T energy transfer. As adsorption is not activated, but desorption is, it is very unlikely that vibrational effects to both processes would be identical and cancel in an experiment that probes the effective sticking coefficient.

During the experiment, the laser is continuously modulated at 3 Hz. The measured partial pressure is directly related to the fraction of the molecular beam that is adsorbed. Therefore, we expect any effect of the vibrational excitation on the sticking probability to be visible in the measured partial pressure at the same modulation frequency. Inspection of the Fourier transform of the measured partial pressure allows us to quantify any effect of the vibrational excitation.

## 3 Results and discussion

### 3.1 RAIR spectra of CO_2_ ice

**FIGURE 2:**
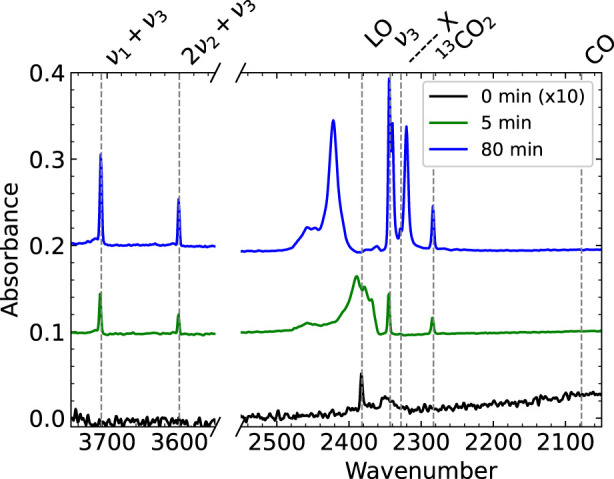
RAIR spectra after dosing CO_2_ from a pure CO_2_ molecular beam for 0–3 mintues (black), 5 min (green) and 80 min (blue). The 0 min spectrum is scaled by a factor of 10. At the start of dosing, only the Longitudinal Optical mode and one absorbance at 2,350 cm^−1^ appear. We assign the peak at 2,350 cm^−1^ to CO_2_ directly physisorbed on the Cu(111) surface. The known peaks for CO_2_ ice from literature are clearly visible in the 5 and 80 min spectra. Note that the apparent absorbance at 2,320 cm^−1^ in the blue spectrum is an artifact discussed in more detail in the Supplementary Information.

We first study the RAIR spectra of the first few layers of physisorbed CO_2_ on the Cu(111) surface. Figure 2 shows three spectra zoomed in on the regions of interest. Outside of these regions, the spectrum shows no features, except for a large set of water absorbances due to the formation of ice on the detector. After dosing for 0–3 min (the time it takes to complete the RAIRS measurement), only two very small peaks are visible, one around 2,350 cm^−1^ and the Longitudinal Optical mode. We assign this peak at 2,350 cm^−1^ to the CO_2_ physisorbed directly on the (uncleaned) Cu(111) surface, as it is the first peak to appear and it becomes masked after dosing many layers of CO_2_. A much narrower and redshifted peak appears in the spectrum taken after dosing for 5 min. This *ν*_3_ CO_2_ ice absorbance and the other absorbances are listed and assigned in Table 1). These peaks continue growing and changing throughout the exposure to the molecular beam. The absorbance of surface-bound CO at 2078 cm^−1^ for (oxidized) Cu(111) (Zhang et al., 2022), that could indicate dissociative CO_2_ adsorption or CO contamination from the background, is absent. Due to the water condensation on the detector, we cannot quantify water contamination in the ice with RAIRS. However, in previous Temperature Programmed Desorption (TPD) experiments, we studied CO and H_2_O desorption and found that water contamination was even less than CO contamination on the sample’s surface. The absorbance suddenly appearing in the final spectrum (blue) at 2,320 cm^−1^ is an artifact. See the Supplementary Information for more detail.

**TABLE 1:** Several peaks in the RAIRS spectrum associated with CO_2_ ice, as found in literature.

Wavenumber	Explanation and references
2,078	CO on Cu(111) (not present) Hollins and Pritchard, (1979); Zhang et al. (2022)
2,283	*ν*_3_ for^13^CO_2_ Sandford and Allamandola, (1990)
2,328	X, Amorphous CO_2_ ice Escribano et al. (2013); Falk, (1987)
2,343	*ν*_3_ Escribano et al. (2013); Sandford and Allamandola, (1990); Falk, (1987)
2,382	Longitudinal Optical mode Escribano et al. (2013); Baratta and Palumbo, (1998)
2,360–2,385	Multilayers, *ν*_3_ coupled to lattice vibrations Ioppolo et al.(2022); Pachecka et al. (2017)
2,385–2,420	Fano line shape Koitaya et al. (2016)
3,601	2*ν*_2_ + *ν*_3_ Sandford and Allamandola, (1990)
3,708	*ν*_1_ + *ν*_3_ Sandford and Allamandola, (1990)

We hypothesize that vibrational excitation of CO_2_ prior to impinging onto the surface may have two effects. Firstly, as discussed before, it may affect the effective sticking probability of CO_2_. Secondly, it may affect the structure of the resulting CO_2_ ice. The ice is known to grow both in amorphous and crystalline phases (Falk, 1987). Dissipation of vibrational energy during the collision or after trapping may locally affect the ice’s growth mode. The introduction of defects would lower the crystallinity of the ice. We investigate our results for clues to either effect.

**FIGURE 3:**
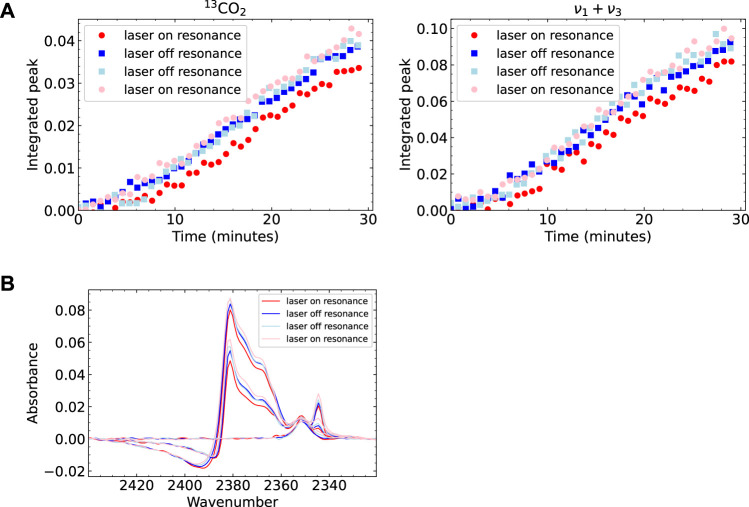
Analyzed RAIRS data for four experiments, of which two had the laser tuned to the CO_2_ resonance frequency. On resonance shown in red (circles), off resonance shown in blue (squares). The experiments were performed in the order as shown in the legend **(A)** Integrals of two peaks in the CO_2_ RAIR spectrum as a function of dosing time **(B)** The large peak in the RAIR spectrum for 3 different points in time during dosing. Note that there is some variation between the measurements which can be seen in both the integral plots and the spectrum plot. The peak growth is slower for the experiments performed later. However, there is no obvious difference between laser on or off resonance. In the bottom figure several features can be seen in the peak. These also seem to be the same for laser on and off resonance, suggesting there is no difference in CO_2_ structure.

We examine the integrals of two peaks in the spectra as a function of time to address a possible influence on the effective sticking probability. We use the peaks at 2,383 cm^−1^ (^13^CO_2_) and 3,708 cm^−1^ (*ν*_1_ + *ν*_3_). These are sharp, isolated peaks that can be integrated accurately and identify two different subsets of molecules. The former is an isotopologue and cannot have been affected by laser excitation. The latter does results from ^12^CO_2_ and may have been affected. Figure 3A shows the integrals for four different experiments performed consecutively. Two of the experiments had the laser tuned to the resonance frequency (red circles), and two experiments had it tuned to a non-resonant frequency (blue squares). The legend (and the lightness of the colors) shows the order in which the measurements were performed. The data show, in general, very similar growth rates. For both absorbances, the earliest experiment lags slightly in comparison to the subsequent measurements. As it happens for both absorbances (one potentially affected by laser excitation and the other surely not), it is most likely the result of small changes in the crystal surface, e.g., cleanliness affecting binding, hence the relative adsorption and desorption rates (see Supplementary Material S1). We do not expect the background pressure of CO_2_ in the vacuum chamber to contribute to the ice growth significantly, as the flux of the molecular beam is approximately two orders of magnitude larger than the background flux. Regardless of the (dominant) origin, the variation in time-dependent absorbance is, apparently, significant in comparison to any potential effect of vibrational excitation on the net sticking.

To address a potential effect of vibrational excitation on the grown CO_2_ ice structure, we study the shape and position of the peaks in the RAIR spectra. Figure 3B shows the spectrum between 2,320 and 2,440 cm^−1^ for the four measurements at three points in time. The visible features here are the peak of CO_2_ on Cu(111) at 2,350 cm^−1^, the *ν*_3_ ice peak at 2,343 cm^−1^, the broad peaks at 2,360–2,385 cm^−1^ and the fano line shape at 2,385–2,420 cm^−1^. As expected from the previous discussion, we find minor variations in peak height between the different measurements. When looking for any spectral change, such as a peak or shoulder that is only present in the data sets where the laser was on resonance, or a shifted peak position, we do not find any such differences. We also looked at changes in the other absorbances outside of the displayed wavenumber range also show no differences between laser ‘on’ and ‘off’ experiments. We conclude that vibrational excitation of the incident molecule does not measurably affect the ice’s IR absorbances, hence the crystallinity of growth at 80 K, nor does the *ν*_3_ vibrational excitation affect the rate of CO_2_ ice growth on the Cu(111) surface significantly.

### 3.2 Sticking probability of CO_2_ on CO_2_ ice

**FIGURE 4:**
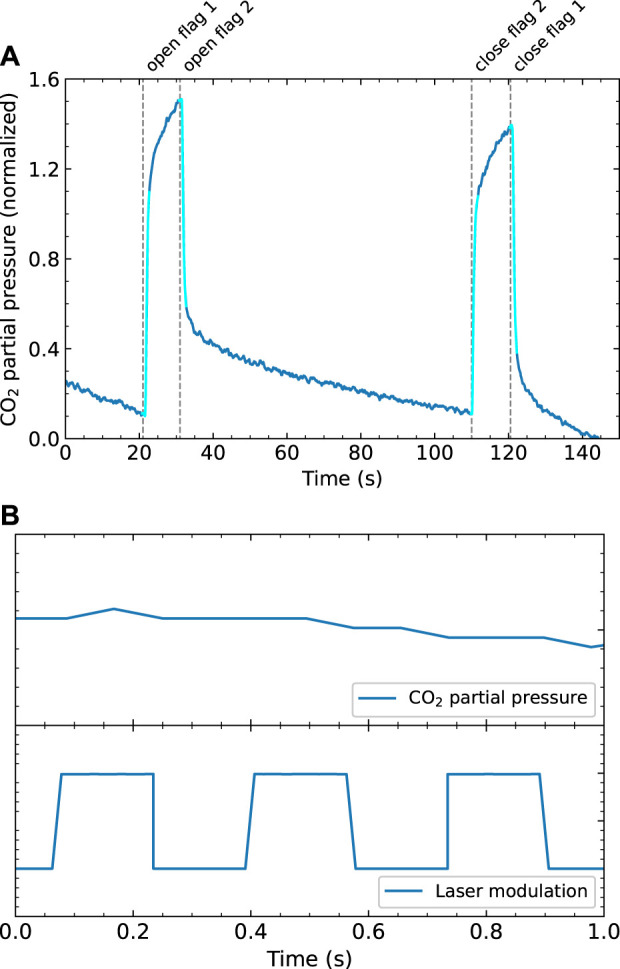
**(A)** An example of a King&Wells experiment of CO_2_ on CO_2_ ice. The signal is normalized to the pressure difference caused by the molecular beam (corresponding to a sticking probability of 1). CO_2_ sticking occurs approximately between 35 and 110 s **(B)** Zoomed in plot of a K&W experiment with the laser modulated at 3 Hz, which shows the measured partial pressure (top) and measured laser modulation (bottom) during 1 s.

Figure 4A shows an example of a King & Wells experiment for CO_2_ adsorption on CO_2_ ice at 80 K after the beam has been on for more than 1 hour. The results are normalized to the partial pressure of the incoming molecular beam, as this pressure change corresponds to a sticking probability of 1. If all molecules from the beam are adsorbed, the partial pressure would be the same as when the beam does not enter the vacuum chamber at all. CO_2_ sticking occurs when the second flag is opened, which is approximately between 35 and 110 s. We use the ratios of the first pressure drop and the initial pressure rise and the second pressure rise and final pressure drop to calculate the overall sticking probability, shown in Figure 4A as the light blue areas. We find values over 0.9. Obtaining absolute values is complicated by the varying effective pumping speed for CO_2_ as evident from the curvature in the time-dependence after opening and closing flags. The effective pumping speed varies as a consequence of the changing CO_2_ partial pressure in the UHV chamber and the cold finger acting as a cyrogenic pump, similar to earlier studies performed on NO sticking (Berenbak et al., 1998). However, in this work we are mainly interested in changes in sticking due to vibrational excitation, which happens fast (at 3 Hz), so we expect no interference of the slowly changing background. Additionally, the effective pumping speed changes slightly when flag 2 is opened or closed, as the molecular beam is scattered from a different location in the vacuum chamber. This may also affect the effective pumping speeds of the turbomolecular pumps and the cryostat, and, hence cause a change in background pressure. During the laser on/off experiment, however, flag 2 is not opened or closed and the data cannot be affected by this potential influence. Figure 4B zooms in on a single second of a King & Wells measurement and the laser modulatation signal at 3 Hz, which is measured simultaneously to verify the modulation frequency. On this time scale, there is no clear switching between two different sticking probabilities.

**FIGURE 5:**
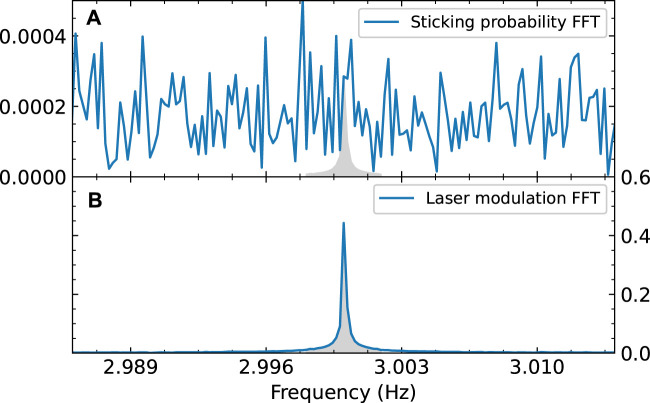
FFT of the measured sticking probability of CO_2_ **(A)** and of the modulation signal of the laser **(B)**. Both are normalized; **(A)** is normalized so the amplitude of the FFT reflects the sticking probability, **(B)** is normalized so an FFT amplitude of 1 corresponds to a square wave with an amplitude of 1. The gray area in **(B)** shows an integral of 1. The modulation frequency of the laser is clearly visible in the FFT spectrum, but in the data it is absent or indistinguishable from the noise. To calculate the integral of the “peak” (shown as the gray area in **(A)**), we assume the same peak shape as in the modulation FFT.

We calculate the Fast Fourier Transform (FFT) of both the measured partial pressure and the laser modulation signal. The data is not always measured at regular intervals. Since the FFT calculation requires a regular sampling rate, we first interpolate the data to a sampling rate of 100 Hz. The absolute values of the FFT are normalized such that they reflect the quantity of interest: for the laser modulation data, this is the amplitude of the measured square wave (which is normalized to 1). For the partial pressure data, it is the amplitude of the square wave normalized to reflect the change in sticking probability. This means that a peak in the sticking probability FFT spectrum with an integral of 1 would correspond to a change in sticking probability of 1 due to laser excitation, or an integral of 0.05 of the peak would correspond to a change in sticking probability of 0.05, *etc.* The results are shown in Figure 5. In the laser modulation FFT spectrum, the peak at 3 Hz is clearly visible, as expected. However, in the FFT spectrum of the sticking probability, it is unclear if there is a peak at 3 Hz. If it is there, it is not clearly distinguishable from the surrounding noise. This implies that if there is any effect of the vibrational excitation on the effective sticking probability, it is below our detection limit.

We can quantify this detection limit to calculate an upper limit for the effect of vibrational excitation of the sticking probability. Due to the finite (and slightly irregular) sampling rate of the measurement, the peak in the FFT spectrum is broadened. Therefore we take the integral of the peak between 2.998 and 3.002 Hz, which is shown as a filled gray area in Figure 5B. For the sticking probability FFT, the peak (if there is any) is not visible, so we have to make some assumptions to calculate its integral. We assume the peak shape is the same as for the laser modulation FFT, because we expect the modulation in the sticking probability to have the same square wave shape as the laser modulation data. We also assume that the value of the FFT at 3.00 Hz is the maximum of this peak. The resulting peak is shown as a gray area in Figure 5A. The integral of the peak, which corresponds to our detection limit for the sticking probability, is calculated to be 6 × 10^−4^ whereas the sticking probability itself is near unity.

The detection limit is the minimum change in sticking probability that we can detect. This does not correspond directly to the minimum effect of vibrational excitation as not all CO_2_ in the beam is vibrationally excited by our laser. Its linewidth allows only excitation of molecules from a particular initial rotational state, and only a fraction of all CO_2_ in the molecular beam is in that initial rotational state. Furthermore, due to limited laser power, less than 100% of the CO_2_ in a particular initial state is excited, even when using rapid adiabatic passage. Finally, spontaneous emission during the 37 cm flight path to the sample after excitation region, also lowers the vibrationally excited fraction of the molecular beam. We have determined the rotational state distribution of the molecular beam and the excited population of the R (4) transition. The methods and results are described elsewhere (Jansen et al., 2023). There, we find that approximately 20% of the CO_2_ molecules are initially in the *J* = 4 state and approximately 80% of these are excited during the experiment. Based on the natural life time 2.4 ms (Gordon et al., 2022) and average velocity of 587 m/s, as determined by TOF, approximately 30% of the excited population is lost via spontaneous emission. Applying these corrections to our detection limit, we find that the upper limit for the change in the CO_2_ sticking probability on CO_2_ ice due to the asymmetric stretch vibration is approximately 5 × 10^−3^.

In comparison to the earlier study on the condensation of CCl_4_ and SF_6_ onto their ices by Sibener & Lee (Sibener and Lee, 1994) our results are somewhat surprising. Although their study was not state-resolved, using different oven temperatures to create a molecular beam in combination with a velocity selector, they convincingly showed that, at low translational energy, molecular sticking is inhibited by the excitation of internal modes. This inhibiting effect was argued to result from (V, R) → T energy transfer. The velocity dependence of this effect, however, also showed that the inhibition decreased with increasing kinetic energy. This was argued to result from a loss of relevance when kinetic energy is too high to be accommodated in the collision for trapping and thermalization to occur. At similar speeds for our experiments and theirs (exceeding 500 m/s), the influence of internal energy on condensation was lost. Hence, although our experiments clearly show that under current conditions potential V (*ν*_3_) → T energy transfer is not affecting the effective sticking probability through enhanced direct scattering or more efficient desorption, it may be of relevance at considerably lower collision energies. In that case, a difference may be more readily detected by monitoring the scattered fraction as it constitutes the minority, as done by Sibener and Lee.

## 4 Summary

Using a combination of molecular beam techniques, state-resolved excitation, and RAIRS detection, we have investigated the possible influences of vibrational excitation on both the sticking of CO_2_ on CO_2_ ice and the structure of the resulting ice on a cryogenically cooled Cu sample. We find no evidence of an influence of the *ν*_3_ mode on either possible effect within our detection limits at an incident velocity of 587 m/s. With regards to the ice structure are our results consistent with the results of Ioppolo et al. (Ioppolo et al., 2022) for crystalline CO_2_ ice. Neither vibrational excitation of molecules prior to impact nor when already in the ice seems to affect the ice structure significantly. With regards to the sticking probability we find that our detection limit supports that, in comparison to the measured average sticking probability for all states present in the beam, it is safe to assume that a single quantum in the *ν*_3_ mode is of no relevant influence to the effective sticking probability. This may result from a fortuitous cancellation of effects on the adsorption vs. direct scattering and desorption rates for the excited state, but this seems unlikely. The result, therefore, expands upon the conclusion from an earlier study that used state-resolved detection of CO_2_ molecules desorbing from its ice: even vibrational energies exceeding the desorption energy seem not relevant to the growth or evaporation of CO_2_ ice.

## Data Availability

The raw data supporting the conclusion of this article will be made available by the authors, without undue reservation.
