# Corrigendum: State-resolved studies of CO_2_ sticking to CO_2_ ice

**DOI:** 10.3389/fchem.2024.1361255

**Published:** 2024-01-17

**Authors:** Charlotte Jansen, Ludo B. F. Juurlink

**Affiliations:** Leiden Institute of Chemistry, Leiden University, Leiden, Netherlands

**Keywords:** CO_2_, nu3, state resolved, molecular beam, condensation

In the published article, there was an error in Figure 5 as published. The wrong dataset was used, see text correction. The corrected Figure 5 and its caption appear below.

**FIGURE 5:**
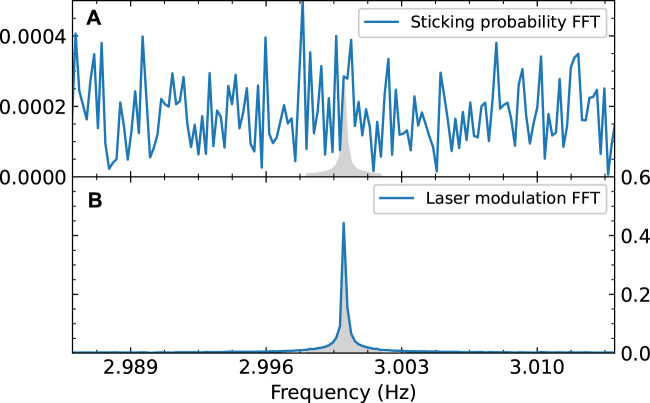
FFT of the measured sticking probability of CO_2_ **(A)** and of the modulation signal of the laser **(B)**. Both are normalized; **(A)** is normalized so the amplitude of the FFT reflects the sticking probability, **(B)** is normalized so an FFT amplitude of 1 corresponds to a square wave with an amplitude of 1. The gray area in **(B)** shows an integral of 1. The modulation frequency of the laser is clearly visible in the FFT spectrum, but in the data it is absent or indistinguishable from the noise. To calculate the integral of the “peak” (shown as the gray area in **(A)**), we assume the same peak shape as in the modulation FFT.

In the published article, there was an error. The King and Wells measurements were done with a cold cathode pressure gauge (Pfeiffer IKR 270). This pressure gauge was found to have a relatively slow response time. A test measurement showed that the sensitivity at 3 Hz is approximately 300 times lower than the maximum sensitivity at 0 Hz. As our measurements were done with a modulating input (laser excitation) at 3 Hz, the upper limit for the effect of laser excitation on sticking probability was overestimated by a factor of 300. However, we have another dataset of the same measurement, but measured with a quadrupole mass spectrometer (QMS, Pfeiffer QMA 200). The change in sensitivity of the QMS between 0 Hz and 3 Hz is negligible. While the overall noise level of the QMS is higher than that of the pressure gauge, it is still much more sensitive at 3 Hz and it is therefore better to use the QMS data.

A correction has been made to **Abstract**. This sentence previously stated:

“Based on our detection limit, we quantify the weighted average sticking probability at approximately 0.9 and the difference between the state-resolved and weighted average sticking probability as below 0.03%.”

The corrected sentence appears below:

“Based on our detection limit, we quantify the weighted average sticking probability at approximately 0.9 and the difference between the state-resolved and weighted average sticking probability as below 0.5%.”

A correction has been made to **Experimental**, 8. This sentence previously stated:

“Hence, we measure the sticking probability of CO_2_ onto CO_2_ ice at 80 K with a modulated version of the King and Wells method (**King and Wells, 1972**) and a cold cathode pressure gauge (Pfeiffer IKR 270). The absolute pressure changes in the UHV chamber are dominated by the molecular beam, which consists (nearly) only of CO_2_. As the ion gauge signal yields considerably better signal-to-noise than our QMS tuned to m/z 44, and it allows for higher detection frequency, it is easier to detect small differences in the sticking probability. The use of an ion gauge instead of a QMS was inspired by prior O_2_ state-resolved measurements (**Kurahashi, 2016**; **Cao et al., 2019**).”

The corrected sentence appears below:

“Hence, we measure the sticking probability of CO_2_ onto CO_2_ ice at 80 K with a modulated version of the King and Wells method (**King and Wells, 1972**) and a quadrupole mass spectrometer (QMS, Pfeiffer QMA 200) tuned to m/z 44.”

A correction has been made to **Experimental**, 9. This sentence previously stated:

“This causes a rise in the CO_2_ partial pressure in the vacuum chamber, which is continuously monitored with the pressure gauge.”

The corrected sentence appears below:

“This causes a rise in the CO_2_ partial pressure in the vacuum chamber, which is continuously monitored with the QMS.”

A correction has been made to **Results and discussion**, *Sticking probability of CO*_*2*_ *on CO*_*2*_ *ice, 3*. This sentence previously stated:

“The integral of the peak, which corresponds to our detection limit for the sticking probability, is calculated to be 3 × 10^−5^ whereas the sticking probability itself is near unity.”

The corrected sentence appears below:

“The integral of the peak, which corresponds to our detection limit for the sticking probability, is calculated to be 6 × 10^−4^ whereas the sticking probability itself is near unity.”

A correction has been made to **Results and discussion**, *Sticking probability of CO*_*2*_ *on CO*_*2*_ *ice*, 4. This sentence previously stated:

“Applying these corrections to our detection limit, we find that the upper limit for the change in the CO_2_ sticking probability on CO_2_ ice due to the asymmetric stretch vibration is approximately 3 × 10^−4^.”

The corrected sentence appears below:

“Applying these corrections to our detection limit, we find that the upper limit for the change in the CO_2_ sticking probability on CO_2_ ice due to the asymmetric stretch vibration is approximately 5 × 10^−3^.”

The authors apologize for these errors and state that this does not change the scientific conclusions of the article in any way. The original article has been updated.

