# Supplementary material for: State-resolved studies of CO2 sticking to CO2 ice
Source: Front Chem. 2023 Aug 24;11:1250711. doi: 10.3389/fchem.2023.1250711 (PMC10483124; doi:10.3389/fchem.2023.1250711)
Supplement: Supplementary file 1 [file DataSheet1.PDF]

Supplementary Material

1 EFFECT OF SURFACE OXIDATION ON CO_2 ICE GROWTH

The sticking measurements were done on CO_2 ice grown on an uncleaned crystal surface at 80 K. Surface oxidation may change the structure of the CO_2 ice, especially in the first few layers. Figure S1 shows the RAIR spectra of several experiments. The top left shows spectra of the ice used for the sticking measurements. The top right graph shows spectra taken during the first 14 minutes of CO_2 dosing onto a well-cleaned Cu(111) surface. The surface was cleaned with sputter-anneal cycles as described previously.[1] The other two graphs show spectra taken in subsequent measurements, where the crystal was only flashed to 300 K between each measurement, but not cleaned with additional sputter-anneal cycles. Lack of simultaneously present LEED and AES optics prevents us from quantifying the level of oxidation of the surface and/or adsorption of other contaminants. For reason of increased use of the surface without cleaning we label these spectra only qualitatively as ”slightly dirty” and ”dirty”. There are some differences between the spectra, mainly near 2380 cm^{-1}, where the ”dirty” surface shows significant absorbance. We do not know the origin of this absorbance, but speculate that it results from interaction of CO_2 with an oxidic Cu surface. Oxidation is known to occur patchwise on Cu(111).[2] The spectrum from the sticking measurement shows the same absorbance after 5 minutes of dosing. The absorbance disappears after dosing many layers of CO_2. This suggests that for thick multilayers, the surface oxidation (or other cause for the absorbance) is not likely to significantly affect the crystalline structure of the CO_2 ice.

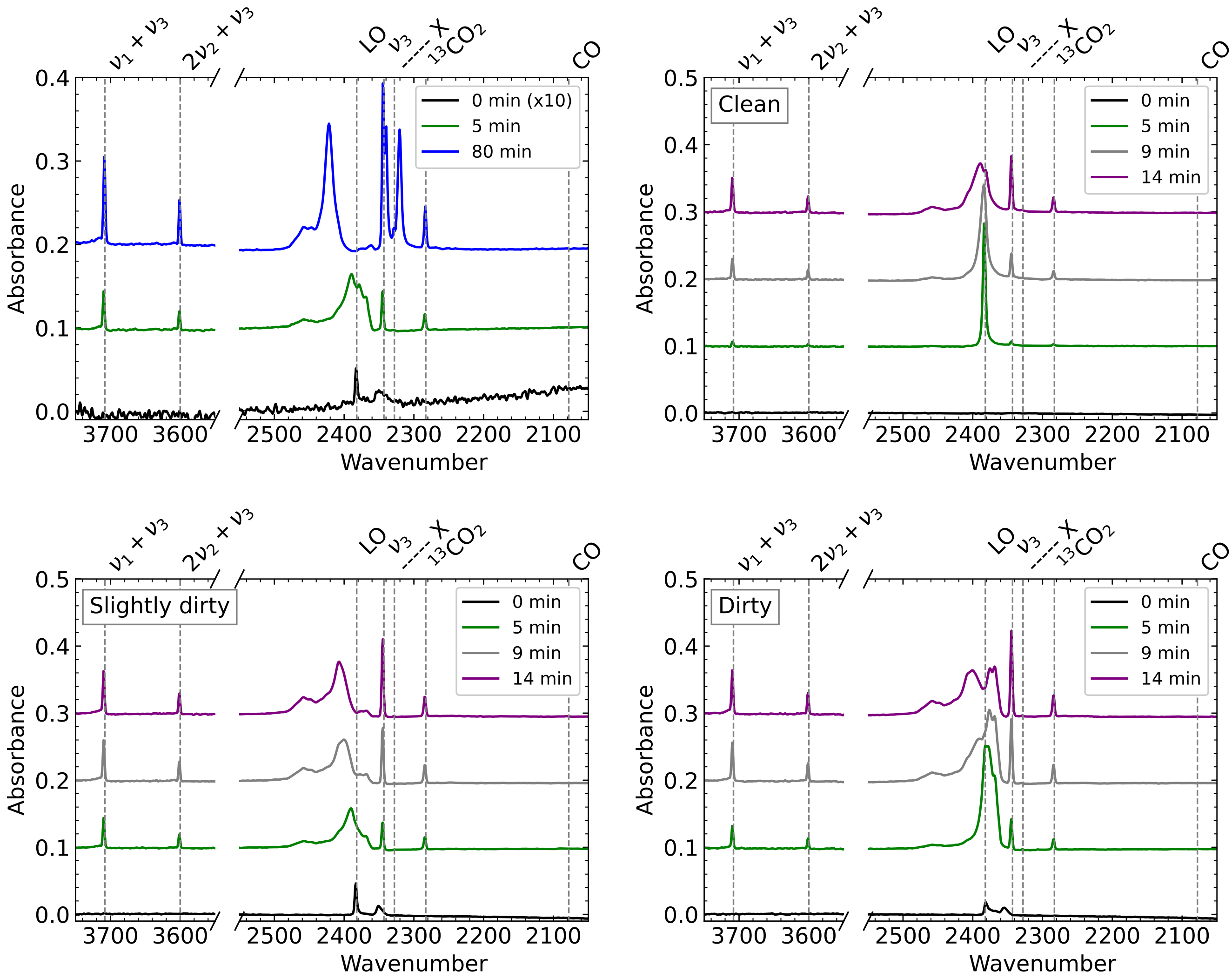

Figure S1. Top left: the RAIR spectra of the CO_2 ice before the sticking measurement. The other graphs show RAIR spectra of the growing CO_2 ice on an increasingly dirty surface.

The top left graph of Figure S1 shows a peak around 2320 cm^{-1}. This is an optical artefact and not an actual CO_2 absorbance. Occasionally, several large peaks of widely varying widths have been found to redshift across spectra, as exemplified in FigureS2. Starting at the third spectrum from the bottom, a well-defined sharp feature suddenly appears at 2340 cm^{-1} - i.e. at the heart of the cluster of CO_2 absorbances. This absorbance redshifts through the CO_2 absorbance window with increased dosing and broadens significantly. It moves out of the spectral range shown here in the eighth spectrum. At the same time, and clearly visible from the fifth spectrum onward, a broad feature enters the shown spectral range at high wavenumbers. Initially, it appears as a bump underneath the combination bands. The bump redshifts through the spectra and becomes increasingly narrow as it approaches the LO mode. We do not understand the origin of this optical effect yet.

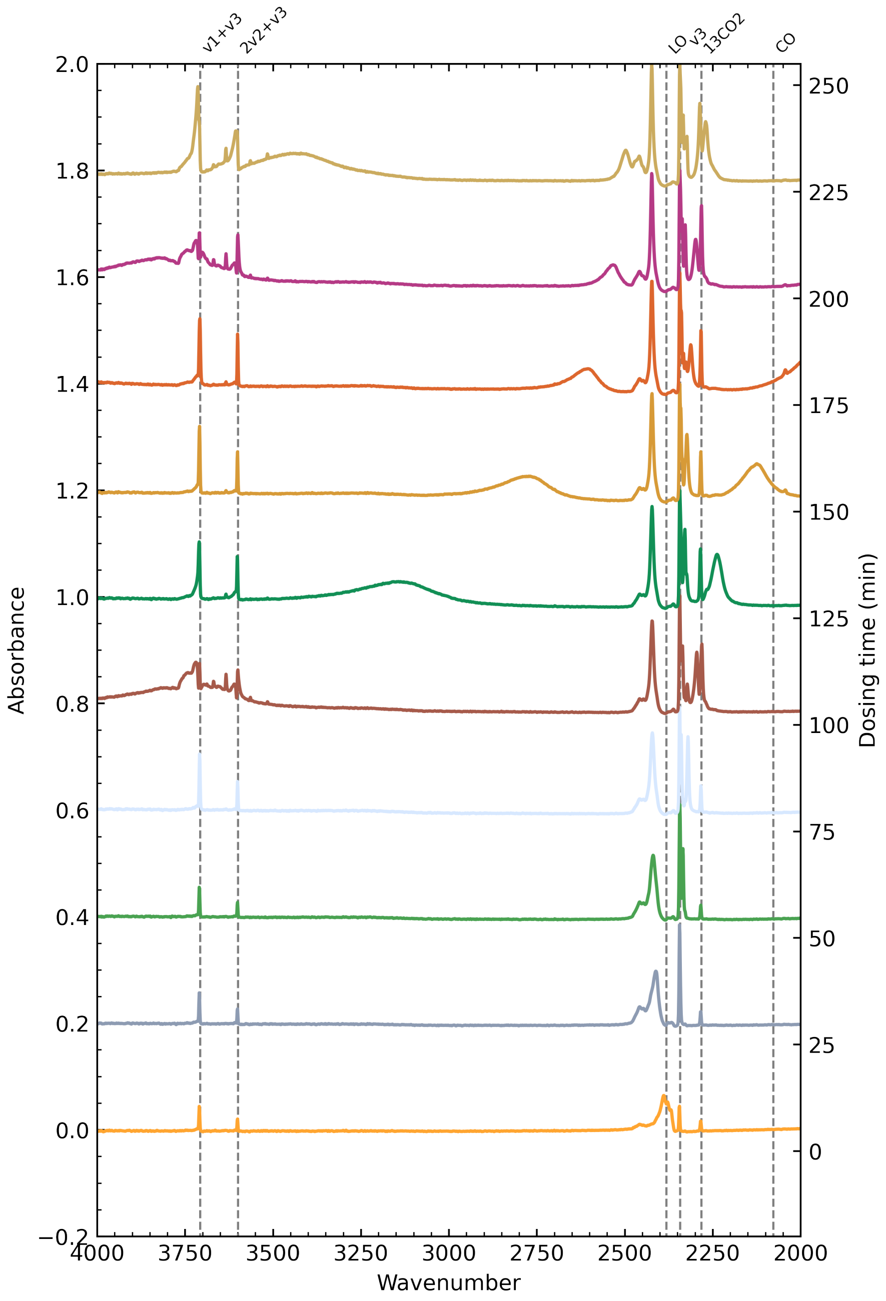

Figure S2. RAIR spectra of the growing CO_2 ice before and during the sticking measurement. There are large peaks shifting through the spectrum. We do not know the origin of these artefacts.

2 TIME OF FLIGHT RESULTS FOR A PURE CO_2 BEAM

Figure S3 shows the velocity distribution of a pure CO_2 molecular beam as measured with our Time of flight experiments (which are described in detail in the next section). The average velocity is determined to be 587 m/s.

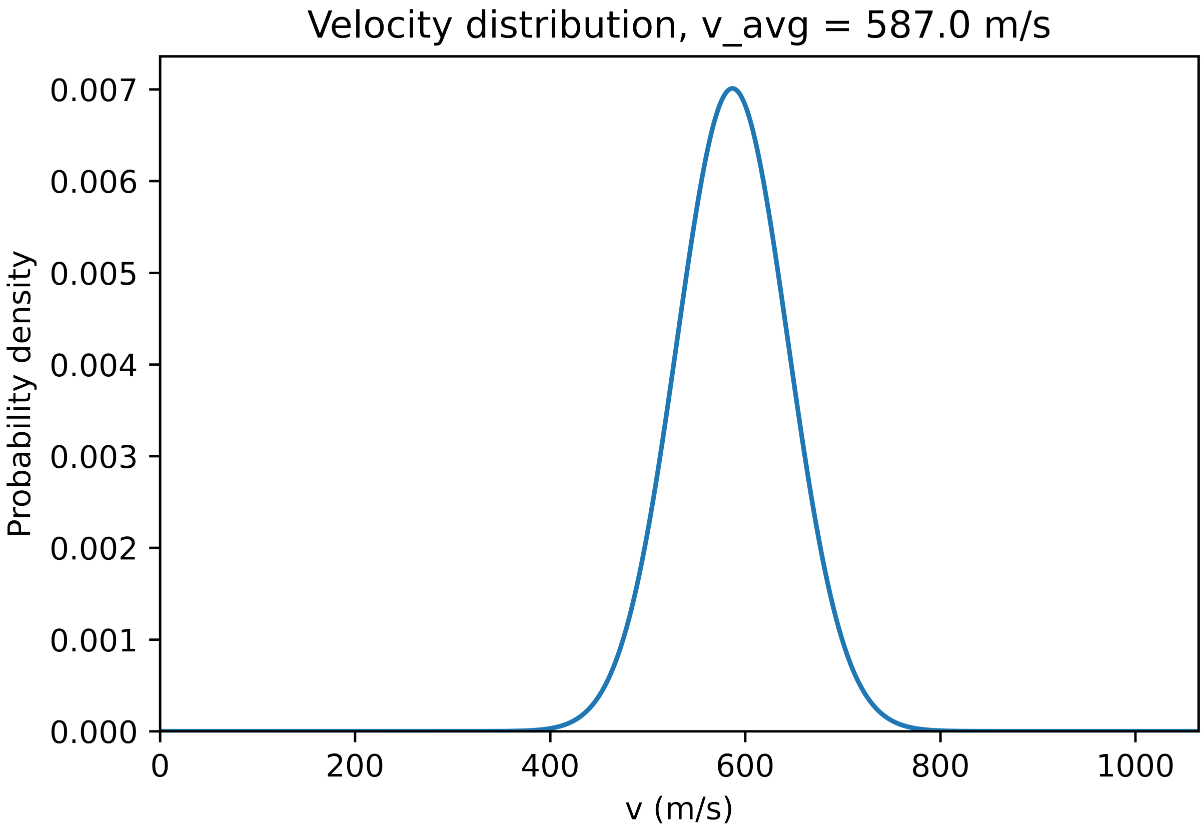

Figure S3. The velocity distribution of a pure CO_2 beam as determined by Time of Flight measurements.

3 TIME OF FLIGHT METHODS

We are often interested in a kinetic energy dependence of sticking and reaction probabilities. An advantage of using a molecular beam is that it has a relatively narrow kinetic energy distribution, which we can measure with Time of Flight (TOF) experiments.

3.1 Theory

The velocity distribution of a molecular beam is given by [3]

$$g(v) = A_v \cdot v^3 \cdot e^{-\left(\frac{v-v_0}{\alpha}\right)^2} \tag{S1}$$

where A_v is an arbitrary scaling parameter, v is the velocity, v_0 the stream velocity and

$$\alpha = \sqrt{\frac{2RT}{M}} \tag{S2}$$

is a measure for the width of the velocity distribution of the molecules in the beam after the expansion, characterized by a temperature T (not to be confused with the nozzle temperature).

3.2 The measurements and data analysis

For a TOF measurement, we use a spinning chopper wheel to generate a short (in the order of microseconds) molecular beam pulse. At the same time, an electronic signal from a photodiode on the chopper wheel triggers the start of the measurement. From here, a QMS facing the molecular beam continuously monitors the partial pressure of the molecule of interest and will therefore be able to measure the flight time of the incoming molecules from the pulse. As there is a velocity distribution in the beam (equation S1), not all molecules will arrive at the same time, and we expect to measure a distribution.

Converting equation S1 to the time domain and taking into account that the QMS is a density sensitive detector, we expect to measure the following distribution with the QMS:

$$f(t) = A \cdot \left(\frac{L}{t - t_s}\right)^4 \cdot e^{-\left(\frac{\frac{L}{t-t_s} - v_0}{\alpha}\right)^2} + B \tag{S3}$$

where A is again an arbitrary scaling parameter, B is the background signal of the QMS, L is the flight path length and $t - t_s$ is the molecules' neutral flight time: t is the measured time and t_s the sum of any time delays in the system.

In order to be able to resolve the shape of this arrival time distribution, we count pulses of the QMS channeltron in windows of 0.5 μs. This results in very small measured signals, so this process is repeated many times by triggering a new measurement when a new pulse is generated by the chopper wheel. All of these measurements are added up until we have sufficient signal to analyze the data, which is done by fitting the data with equation S3 and explained in more detail later.

This TOF measurement is done with the QMS at several different positions (which is useful later in the data analysis). An example of the measured data from a CO_2 + He beam is shown in figure S4, with the datasets for QMS different positions in different colors. Note that the peaks of the different positions are slightly shifted in time, due to the change in flight path length. The top panel shows the He TOF data, the bottom panel shows the CO_2 TOF data. Note that the CO_2 molecules arrive later than the He molecules, indicating velocity slip in the molecular beam.

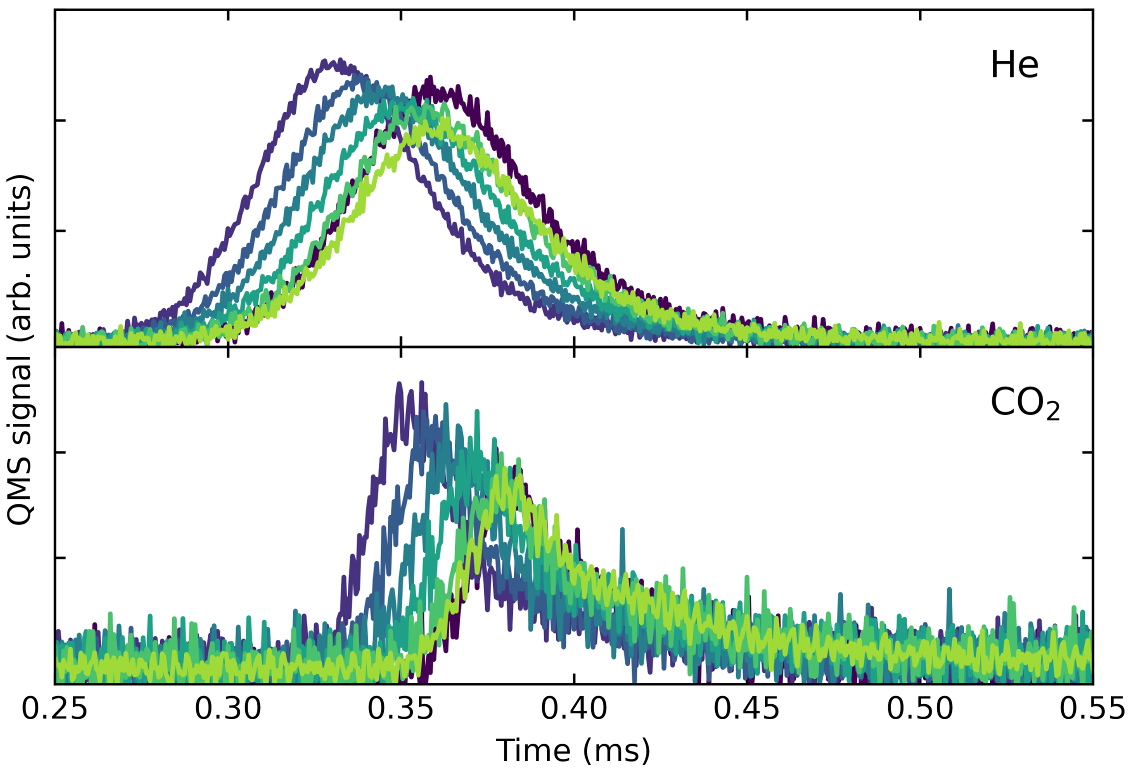

Figure S4. Raw data of a TOF measurement of a mixed molecular beam of CO_2 and He. The different colors show the datasets of the measurement at different QMS positions.

Equation S3 shows the expected distribution of arrival times of molecules in an infinitely short pulse of the molecular beam, as measured by a QMS. In reality, the molecular beam pulse is not infinitely short, but has a certain shape due to the width of the slit in the rotating chopper, as well as the size of the molecular beam itself. Figure S5a shows the shape of the molecular beam pulse, resulting from a rectangular slit moving over a circular beam profile.

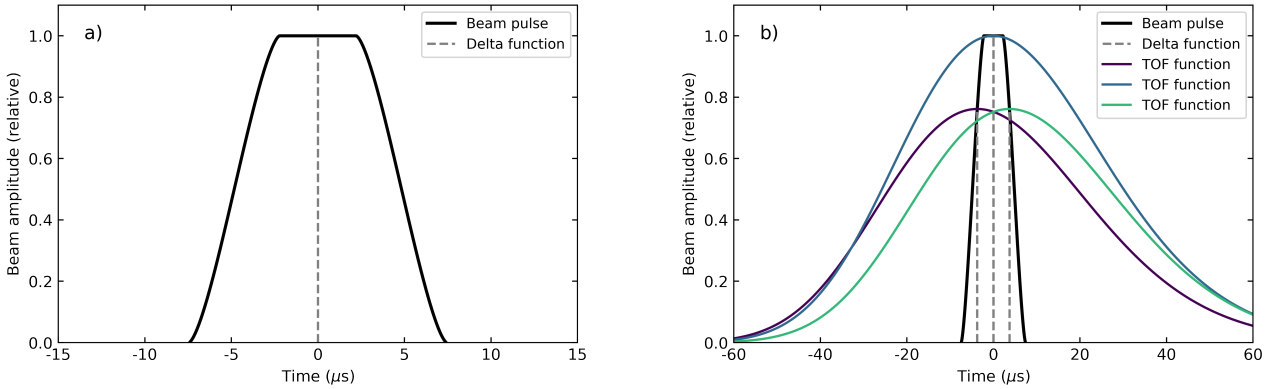

Figure S5. a) The shape of the molecular beam pulse, due to the finite width of the chopper slit. b) The incorporation of the convolution into the data analysis, shown for n=3.

As a result, the actual measured TOF signal is a convolution of equation S3 and the profile of the pulse. We include this in the fitting procedure by fitting the data with a sum of n times equation S3, each shifted by a unique time difference and scaled according to the pulse profile. This is illustrated in figure S5b for n=3. For the data analysis, we typically use n=15. Note that the pulse width is relatively small compared to the width of the expected arrival time distribution.

3.3 Fitting procedure details

The fit parameters are A, B, L, t_s, v_0 and α. L is known, but is included because a precise measurement of L in the system is difficult, and it depends on the exact nozzle position. We have five datasets per experiment, for six different QMS positions (the first and last measurement are typically done at the same position). Since the parameters t_s, v_0 and α are independent of flight path length, they are expected to be the same for each measurement. We make sure these are kept the same for the fit of each dataset. Although L varies between measurements, we know exactly how much it varies. By taking this into account we can also fit a shared L value for all measurements (its value corresponding to L at position 0, and correcting for the difference in position per dataset). A and B depend on the sensitivity of the QMS and background pressure and can vary between measurements. Furthermore, their physical meaning (beam intensity, background pressure) is not of interest in this experiment. Therefore we fit these two parameters separately for each dataset. This results in a set of fitting parameters that consists of one L, t_s, v_0 and α, and seven different A and B parameters. The fit is done on the entire set of seven datasets simultaneously.

t_s includes all mechanisms in the measurement that affect the measured flight time, such as electronic delays in the mass spectrometer signal or trigger, the fact that the trigger is the edge of the chopper wheel slot and not the center and the additional flight time of the molecules after ionization in the mass spectrometer. Although it is possible to estimate some of these delays separately, the error margins are relatively big. Therefore they are all combined into a single fit parameter. The initial guess for t_s is estimated by determining the most probable flight time of the molecules (corresponding to the maximum of the TOF peaks) for all QMS positions by fitting with a simple gaussian function, and extrapolating the results to a path length of 0. Since the molecular flight time should be 0 at this position, the resulting extrapolated "most probable flight time" is an indication of the delay time t_s.

The result of a fit is shown in figure S6, for the same CO_2 + He beam as mentioned in figure S4. The fit for the He molecules is quite good. However, it is off for the CO_2 molecules. This may be caused by the velocity distribution changing as a result of the velocity slip.

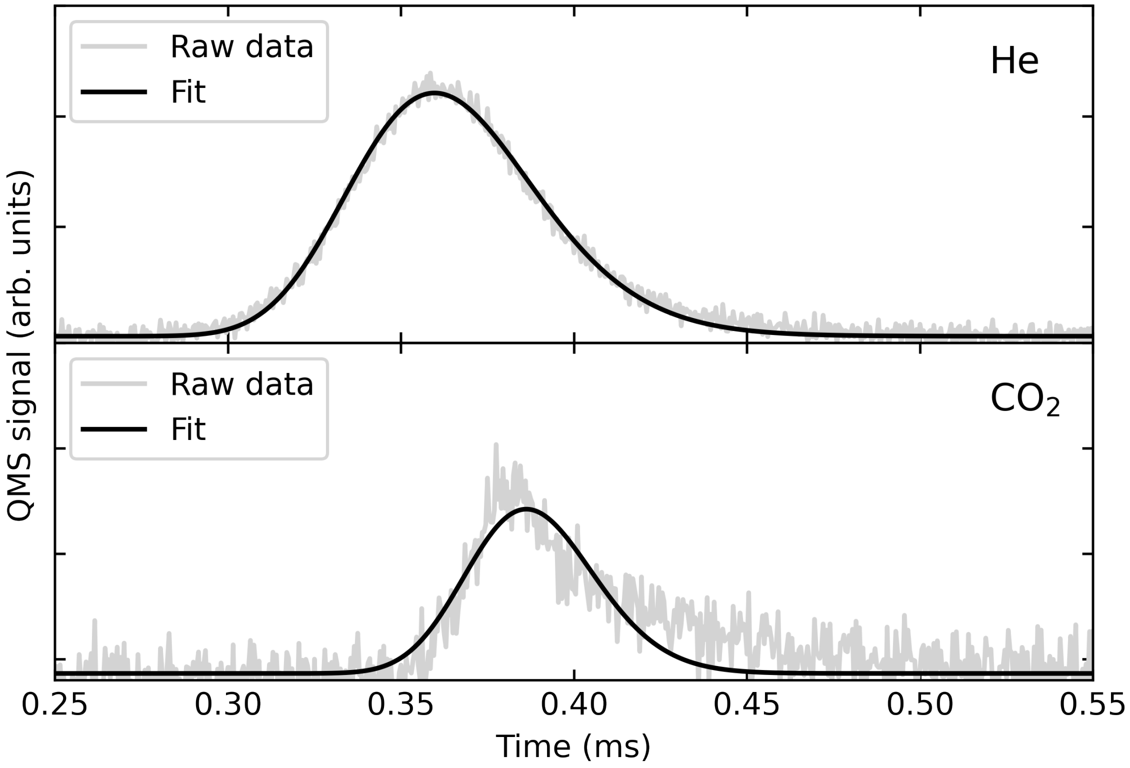

Figure S6. The result of the fitting procedure for a CO_2 + He beam. The fit for the He molecules is quite good, but for the CO_2 molecules the shape is off.

REFERENCES

[1]Diyu Zhang, Charlotte Jansen, Aart W. Kleyn, and Ludo B. F. Juurlink. Adsorption dynamics of O $_2$ on Cu(111): A supersonic molecular beam study. *Physical Chemistry Chemical Physics*, 25(21):14862–14868, 2023.

[2]Diyu Zhang, Charlotte Jansen, Otto T. Berg, Joost M. Bakker, Jörg Meyer, Aart W. Kleyn, and Ludo B. F. Juurlink. RAIRS Characterization of CO and O Coadsorption on Cu(111). *The Journal of Physical Chemistry C*, 126(31):13114–13121, August 2022.

[3]DJ Auerbach. Atomic and molecular beam methods. *by G. Scoles, Oxford Univ. Press, New York*, 1:362, 1988.
